# Linking New Information to a Reactivated Memory Requires Consolidation and Not Reconsolidation Mechanisms

**DOI:** 10.1371/journal.pbio.0030293

**Published:** 2005-08-23

**Authors:** Sophie Tronel, Maria H Milekic, Cristina M Alberini

**Affiliations:** **1**Department of Neuroscience, Mount Sinai School of Medicine, New York, New York, United States of America; Boston University, Boston, MassachusettsUnited States of America

## Abstract

A new memory is initially labile and becomes stabilized through a process of consolidation, which depends on gene expression. Stable memories, however, can again become labile if reactivated by recall and require another phase of protein synthesis in order to be maintained. This process is known as reconsolidation. The functional significance of the labile phase of reconsolidation is unknown; one hypothesis proposes that it is required to link new information with reactivated memories. Reconsolidation is distinct from the initial consolidation, and one distinction is that the requirement for specific proteins or general protein synthesis during the two processes occurs in different brain areas. Here, we identified an anatomically distinctive molecular requirement that doubly dissociates consolidation from reconsolidation of an inhibitory avoidance memory. We then used this requirement to investigate whether reconsolidation and consolidation are involved in linking new information with reactivated memories. In contrast to what the hypothesis predicted, we found that reconsolidation does not contribute to the formation of an association between new and reactivated information. Instead, it recruits mechanisms similar to those underlying consolidation of a new memory. Thus, linking new information to a reactivated memory is mediated by consolidation and not reconsolidation mechanisms.

## Abstract 2

The association of new information with an established memory is mediated through a consolidation mechanism, leaving the original memory intact.

## Introduction

Memory is a dynamic process. A new memory is initially labile and becomes stabilized over time through a process of *consolidation* [1,2]. This process depends on an initial phase of RNA and protein synthesis that has been characterized in several different species and with different types of memories [3–5]. Once stabilized, memory is not permanently fixed and can again become labile if reactivated by recall [6–9]. Indeed, memory is disrupted if a number of interfering events or pharmacological treatments, including protein synthesis inhibitors, are administered during the post-reactivation labile phase. Thus, it has been proposed that this labile, protein synthesis-dependent phase is required to *reconsolidate* the reactivated memory [6,7,9]. However, the reasons why a reactivated memory becomes labile and requires protein synthesis remain to be understood. One hypothesis proposes that the labile state of the reconsolidation process allows new information to be associated with established and reactivated memories [6].

Although some disagreement remains [10], many studies have demonstrated that memory consolidation and reconsolidation have distinct molecular requirements [11–13]. In particular, it has been shown that the two processes have anatomically distinct requirements for specific proteins and protein synthesis in general [11,12]. For example, inhibitory avoidance (IA) memory, in which the animals learn to avoid a context previously associated with a shock, is disrupted by protein synthesis inhibitors administered systemically immediately after reactivation suggesting that, like many other types of memories, IA undergoes reconsolidation [14]. However, the consolidation, but not reconsolidation, of IA memory requires protein synthesis and the function of the transcription factor CCAAT enhancer binding protein β (C/EBPβ) in the hippocampus [14–16], indicating that this region is differentially involved in the two processes. These results also imply that the protein synthesis necessary for the reconsolidation process must occur in brain regions outside the hippocampus. Indeed, in the present study, we report that IA reconsolidation, but not consolidation, requires C/EBPβ in the amygdala. Thus, the C/EBPβ requirement that differentially occurs in the amygdala for reconsolidation and in the hippocampus for consolidation can be utilized to doubly dissociate these two processes.

A paradigm that is appropriate for investigating how new and reactivated information becomes associated is second-order conditioning. Whereas a first-order, classical Pavlovian conditioning involves the formation of an association between the representations of the stimuli paired during training (pairing between a conditioned stimulus [CS] with an unconditioned stimulus [US]), a second-order conditioning promotes the formation of associations between the second CS (S2) and the conditioned response elicited by the first CS (S1) [17,18]. Thus, the stimulus-response learning that occurs during a second-order conditioning represents the formation of an association between new (S2) and reactivated information (memory of S1-US), which makes this paradigm proper for investigating the mechanisms involved in linking new and reactivated information. Here we used the IA task, modified to a second-order conditioning paradigm, and the anatomically distinct C/EBPβ requirements to investigate whether, as hypothesized, the process of reconsolidation, induced by memory reactivation, is utilized to link new learning with already established and reactivated memories.

## Results

### Both the New Information and the Reactivated Memory are Long-Lasting

Using the IA task modified to a second-order conditioning paradigm as described below, we determine (1) whether reactivation of an established memory allows the formation of a new association between the recalled memory and new information, (2) whether the formation of the new association is mediated by reconsolidation or consolidation mechanisms, and finally, (3) whether the formation of the new association interferes with the stability and the reconsolidation of the old memory.

Rats were placed on one side of an IA apparatus (context A), and 2 s later a light was switched on (visual CS). After 10 s the door opened, allowing the rats to enter the dark compartment where they received a foot shock (see Materials and Methods). Rats were divided into three groups 48 h later (Figure 1A). One group underwent a reactivation trial consisting of re-exposure to the light for 90 s, but in a new context (reactivated in B). The other two groups were used as controls: one group was exposed to only context B for 90 s without light (exposed to B), and the other remained in the home cage (non-reactivated). All groups were divided into two subgroups 48 h later; one was tested in context B and the other in context A. A one-way analysis of variance (ANOVA) that compared the mean latencies to dark compartment entry among the groups revealed a significant effect (F_5, 66_ = 11.76, *p* < 0.0001). As shown in Figure 1A, rats that underwent memory reactivation by the light in context B developed a strong memory for context B, compared with both control groups that had virtually no memory. A Newman-Keuls post hoc test confirmed that retention of the group that underwent memory reactivation by the light in context B (369.66 ± 64.81 s) was significantly higher than that of both control groups tested in context B (exposed to B: 69.66 ± 28.29 s and non-reactivated: 106.25 ± 49.76 s, *p* < 0.01). In contrast, independent from the second behavioral experience received in context B, all groups tested in context A had strong retention and there were no significant differences among the groups (reactivated in B: 334.25 ± 55.03 s, exposed to B: 438.25 ± 49.36 s and non-reactivated: 466.91 ± 41.26 s). Interestingly, the new memory for context B was as strong as the memory for context A.

**Figure 1:**
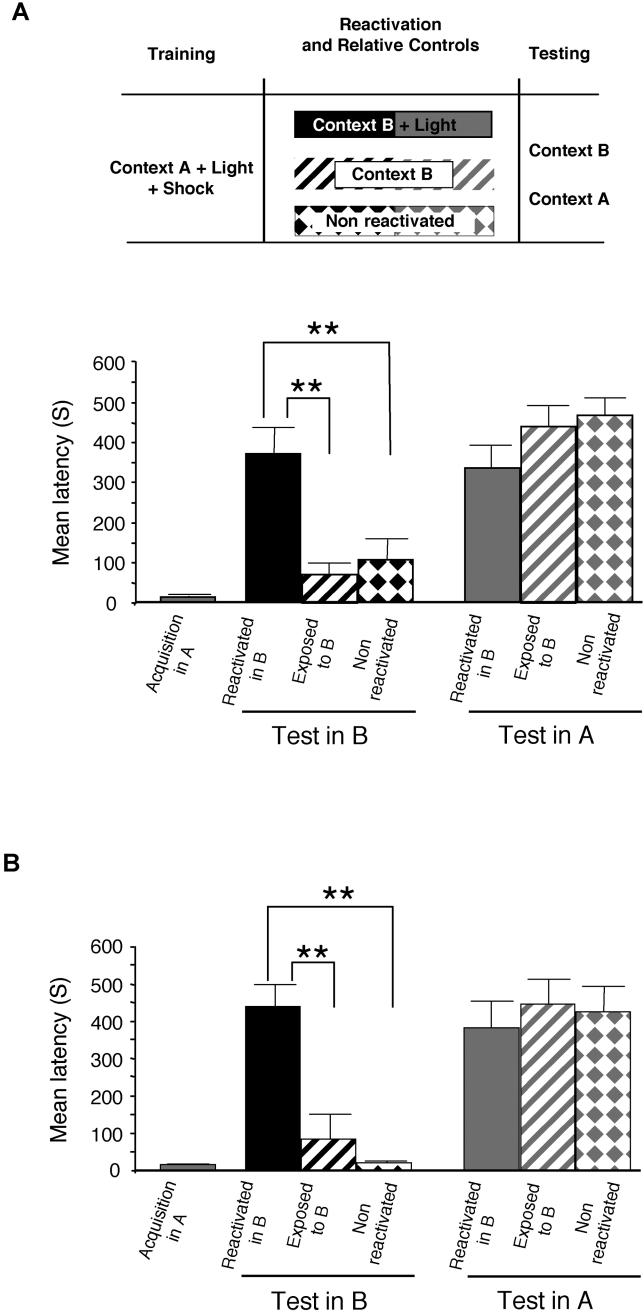
Associations Are Formed between New Information and a Reactivated Memory Both the original and the new memory are long-lasting. Latency to enter the shock chamber was taken as a measure of memory acquisition and retention. (A) Rats trained in context A (*n* = 72) that received memory recall by visual CS in context B developed a strong fear memory for context B (*n* = 12), while both control groups (rats exposed to context B without reactivation and non-reactivated; *n* = 12 per group) had no memory for context B (** = *p* < 0.01). Rats that underwent the same training and reactivation protocol but were tested in context A (*n* = 12) showed retention levels similar to those of both control groups (*n* = 12 per group). Latencies to enter either context A or B were similar, suggesting that the new memory is as strong as the old one. (B) All groups (*n* = 8 per group) were re-tested 1 wk after the first test. Similar results were obtained.

To determine whether the memory for context B is long-lasting, all groups were retested 1 wk later and similar results were found (Figure 1B). A one-way ANOVA revealed a significant difference between groups at the retention test (F_5,42_ = 10.50; *p* < 0.0001). A Newman-Keuls post hoc test showed that the significantly different latencies found 48 h after reactivation were maintained 1 wk later, indicating that the newly formed association is long-lasting (latencies of rats for context B, reactivated in B: 438.25 ± 57.90 s; exposed to B: 83.87 ± 65.22 s; non-reactivated: 20.25 ± 2.76 s. Latencies of rats for context A, reactivated in B: 381.00 ± 71.56 s; exposed to B: 446.25 ± 66.61 s; non-reactivated: 426.00 ± 65.50 s).

Thus, together, these results showed that a long-lasting association is formed between new information and a reactivated IA memory. In other words, a new association is made between the new context B (S2) and the fear that was originally elicited by the light in context A (S1) paired with the shock (US).

### Both the New Association and the Reactivated IA Memory Are Disrupted by Systemic Inhibition of Protein Synthesis

We next determined the effect of protein synthesis inhibitors injected systemically after reactivation in context B on both the new and the reactivated memory. Rats were trained in context A and reactivated in context B, as described above. Immediately after reactivation they received a systemic injection of either anisomycin or vehicle solution, and 1 wk later were tested in context B and then in context A (Figure 2). A two-way ANOVA showed an effect of treatment (F_2,21_ = 18.69; *p* < 0.0001), context of testing (F_1,21_ = 12.41; *p* < 0.01) and a significant interaction (F_2,21_ = 9.39; *p* < 0.01). A Newman-Keuls test revealed that anisomycin disrupted the memory for context B (Aniso: 72.75 ± 24.84 s), compared to vehicle (Veh: 353.75 ± 59.93 s; *p* < 0.01). As expected, the rats that did not undergo reactivation had no retention for context B (19.25 ± 2.88 s**)**.

**Figure 2:**
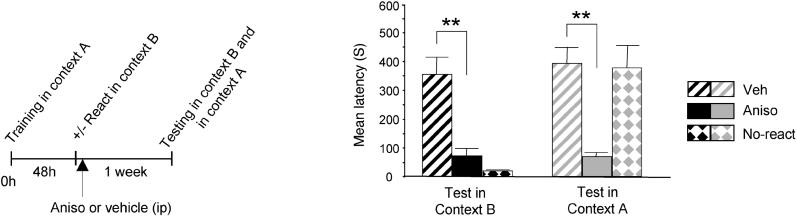
Both the Association Formed between New and Reactivated Information and the Reactivated Original IA Memory Are Disrupted by Systemic Inhibition of Protein Synthesis Latency to enter the shock chamber was taken as a measure of memory retention. Systemic anisomycin injection (Aniso, *n* = 8) immediately after visual CS reactivation in context B induced amnesia for both context A and B compared with vehicle injection (Veh, *n* = 8) (** = *p* < 0.01). Anisomycin did not affect memory of context A in rats that did not receive reactivation (*n* = 8, No-react).

Moreover, in line with previous findings as well as the reconsolidation hypothesis [14,19], systemic administration of anisomycin significantly disrupted the memory of context A, compared with vehicle injection (Aniso: 69.00 ± 14.24 s; Veh: 392.75 ± 56.28 s; *p* < 0.01). The amnesic effect of anisomycin was contingent upon the reactivation event, as rats that did not undergo reactivation and were injected with anisomycin 48 h after training maintained their memory for context A (375.87 ± 77.43 s).

Thus, when a memory is reactivated in the presence of new information and forms new associations, both the new and the original memory undergo a protein-synthesis-dependent phase.

### Consolidation, but Not Reconsolidation, Mechanisms Are Required to Associate New Information with a Reactivated Memory

Because the newly formed association is composed of both new information and the reactivated memory, we investigated the mechanisms that mediate its formation. Does reconsolidation play a major role in this process, or is consolidation also involved?

To address this question, we first set out to determine molecular mechanisms that are differentially engaged in distinct brain regions during either consolidation or reconsolidation of IA.

In a previous study [14], we showed that C/EBPβ is required in the hippocampus for the consolidation, but not the reconsolidation, of IA. Because the amygdala is also known to play a critical role in IA memory formation, we investigated the requirement for C/EBPβ in the amygdala during consolidation and reconsolidation. We found that, in the amygdala, C/EBPβ is required for reconsolidation, but not consolidation, of IA.

In a first set of experiments, rats were trained on IA and five hours later received bilateral injections into the amygdala of either C/EBPβ antisense oligodeoxynucleotide (β-ODN) or, as a control, scrambled oligodeoxynucleotide (SC-ODN). Retention was tested 48 hours after training. As shown in Figure 3A, no significant difference was observed between β-ODN (331.00 ± 59.26 s) and SC-ODN-injected rats (312.08 ± 89.41s), suggesting that, at this time point, C/EBPβ is not essential in the amygdala for IA consolidation.

**Figure 3:**
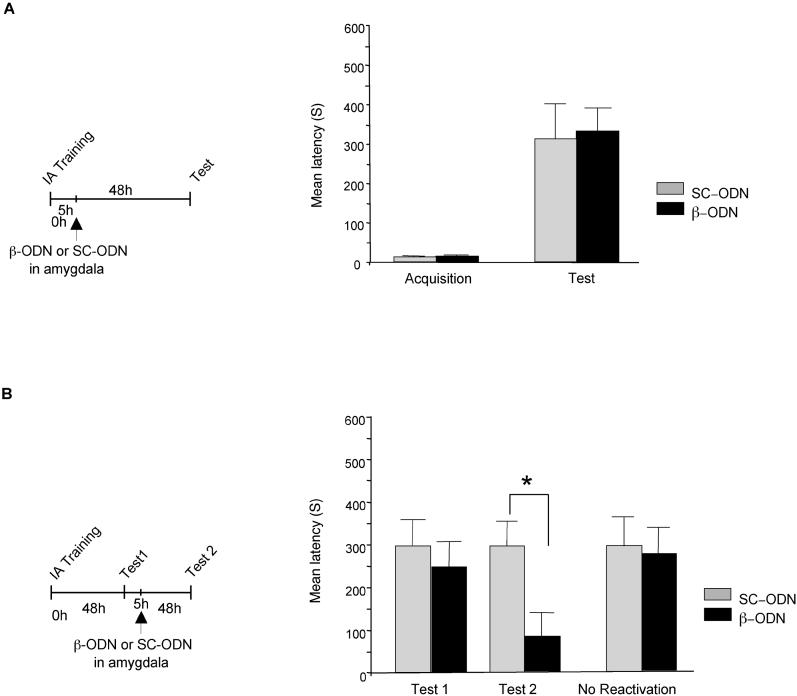
C/EBPβ Is Required in the Amygdala for the Reconsolidation, but Not Consolidation, of IA Memory Latency to enter the shock chamber was taken as a measure of memory acquisition and retention. (A) Bilateral amygdala injections of β-ODN (*n* = 8) 5 h after training had no effect compared to SC-ODN injection (*n* = 8) on IA retention tested 48 h after training. (B) Bilateral amygdala injections of β-ODN 5 h after reactivation (Test 1, *n* = 10) resulted in amnesia at Test 2, compared to SC-ODN injections (*n* = 8, * = *p* < 0.05).

Next, we investigated whether C/EBPβ is required in the amygdala for IA memory reconsolidation. Rats were trained on IA and tested after 48 h (Test 1). 5 h after Test 1, half of the rats received bilateral amygdala injections of β-ODN and the other half of SC-ODN. All animals were again tested for IA memory retention 48 h after Test 1 (Test 2). A two-way ANOVA showed a significant effect of treatment (F_1,46_ = 4.57; *p* < 0.05). A Newman-Keuls post hoc test revealed that, at Test 2, the retention levels of the rats that received β-ODN-injections were significantly lower (83.77 ±17.34 s) than those that received SC-ODN-injections (298.17 ± 61.14 s; *p* < 0.05). All animals had similar latencies at Test 1 (Veh: 297.96 ± 61.28 s; β-ODN: 247.27 ± 51.77 s) (Figure 3B). To test whether the memory disruption caused by β-ODN was contingent upon reactivation, groups of rats were injected with β-ODN or SC-ODN 48 h after training in the absence of Test 1. No significant difference was found in the avoidance latency of animals that received β-ODN (273.85 ± 66.36 s), compared to that of rats injected with SC-ODN (295.66 ± 66.58 s), and both groups showed a strong memory (Figure 3B). Together, these results show that amygdala C/EBPβ is required for the reconsolidation, but not consolidation, of IA.

Hence, at 5 h post-training or post-recall, C/EBPβ is required in the hippocampus for consolidation, but not reconsolidation, and in the amygdala for the reconsolidation, but not consolidation, of IA memory. In other words, the C/EBPβ requirement in either the hippocampus or the amygdala during consolidation or reconsolidation can be used to doubly dissociate the critical contribution of the two processes.

We first tested whether the formation of an association between the new information and the reactivated memory is dependent on amygdala C/EBPβ expression.

Rats were trained in context A, reactivated with the visual CS in context B, and divided in two subgroups. 5 h after reactivation, one group was injected in the amygdala with β-ODN, while the other received SC-ODN injection and served as control. Additional control groups underwent the same training and β-ODN injection procedures, but in the absence of recall. 48 h later, the rats were tested first in context B and then in context A. As shown in Figure 4A, the association between new and reactivated information was not affected by β-ODN injection. However, this treatment disrupted the memory for context A. A two-way ANOVA between treatment (β-ODN /SC-ODN/β-ODN-non-reactivated) and context of testing (A/B) revealed a significant effect of context (F_1, 19_ = 15.68, *p* < 0.001), a significant effect of the treatment (F_2, 19_ = 36.16, *p* < 0.0001), and a significant treatment × context interaction (F_2, 19_ = 81.85, *p* < 0.001). A Newmans-Keuls post hoc test showed that the memory of context A was significantly lower in rats that received β-ODN injection in the amygdala after reactivation (45.14 ± 3.62 s), compared with that of rats that received amygdala SC-ODN-injection, and also that of non-reactivated controls injected with β-ODN (228.62 ± 11.47 s, *p* < 0.01 and 244.28 ± 8.58 s, respectively). In contrast, when the rats were tested in context B, they all showed a strong memory (SC-ODN:164.42 ± 28.88 s and β-ODN: 197.75 ± 10.50 s, respectively). Together, these data indicate that reconsolidation mechanisms are not engaged in the formation of the new association that links new information with a reactivated memory.

**Figure 4:**
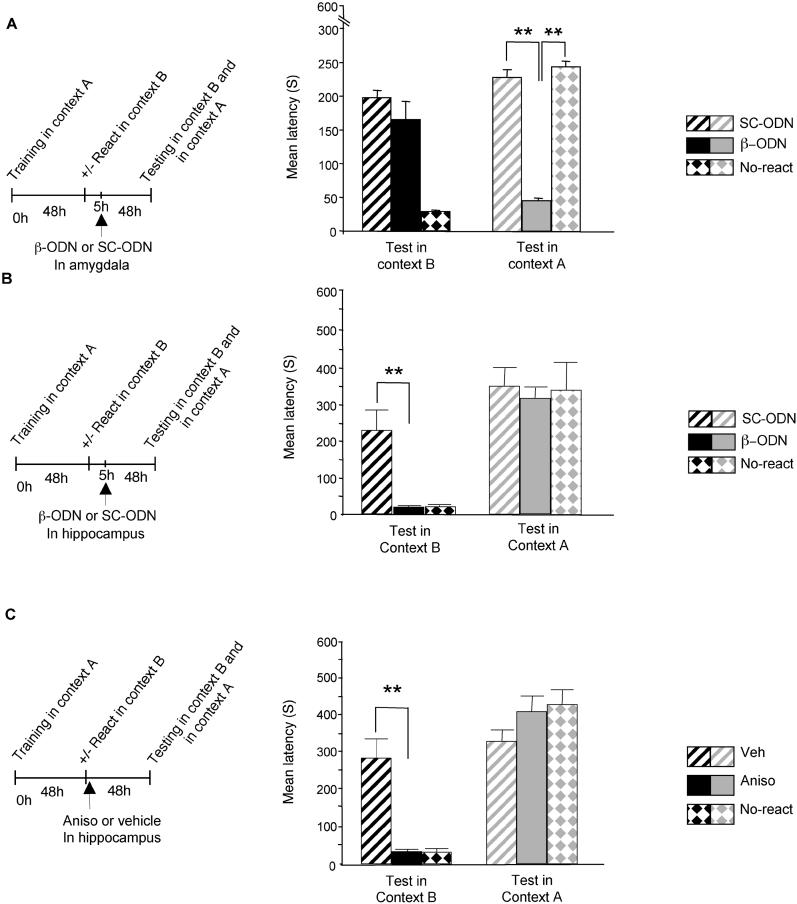
Consolidation, but Not Reconsolidation, Mechanisms Are Required to Associate New Information with a Reactivated Memory Latency to enter the shock chamber was taken as a measure of memory retention. (A) Amygdala injection of β-ODN after memory reactivation disrupted the reactivated memory without affecting the formation of an association between new information and the reactivated memory. β-ODN injection 5 h after reactivation into the amygdala of rats that underwent training in context A and memory reactivation in context B (*n* = 7) had no effect on the memory for context B tested 48 h after reactivation. Memory for context B was similar to that of the control group (*n* = 8) that received SC-ODN injection. In contrast, the same treatment strongly impaired memory of context A, compared to SC-ODN injection (** = *p* < 0.01). Memory of context A was not affected in rats that did not receive reactivation (*n* = 7). (B) Hippocampal injection of β-ODN blocked the formation of the association between new and reactivated information without affecting the stability of the reactivated memory. β-ODN injection 5 h after reactivation into the hippocampi of rats that underwent training in context A and memory reactivation in context B (*n* = 8) significantly impaired the retention for context B 48 h after reactivation, compared with SC-ODN injection (** = *p* < 0.01). β-ODN injection did not affect the memory of context A, which was similar to that of both control groups that received either SC-ODN injection after reactivation or β-ODN injection in the absence of reactivation (*n* = 9 for SC-ODN and *n* = 6 for β-ODN-injected, No-react). (C) Hippocampal injection of anisomycin blocked the formation of an association between new and reactivated information without affecting the reactivated memory. Anisomycin administration (*n* = 8) immediately after memory reactivation in context B significantly impaired memory of context B when tested 48 h later, compared with vehicle injection (*n* = 8; ** = *p* < 0.01). Anisomycin injection did not affect memory of context A (*n* = 8; ** = *p* < 0.01), which was similar to that of vehicle-injected control group (*n* = 8 per group). Anisomycin did not affect memory of context A in rats that did not receive reactivation (*n* = 8, No-react).

We then asked whether the formation of the new association depends on consolidation mechanisms; that is, we tested the requirement for C/EBPβ in the hippocampus.

Rats were trained with IA in context A, and their memory was reactivated in context B as described above. They were divided in two subgroups 5 h after reactivation: one was injected into the hippocampi with β-ODN, while the other received SC-ODN. An additional control group underwent training and β-ODN injection, but in the absence of recall. The rats were tested 48 h later, first in context B and then in context A. As shown in Figure 4B, hippocampal β-ODN injections completely blocked the formation of memory for context B without affecting memory for context A. A two-way ANOVA between treatment (β-ODN /SC-ODN/β-ODN-non-reactivated) and context of testing (A/B) revealed a significant effect of context (F_1, 20_ = 71.28, *p* < 0.0001), and a significant treatment × context interaction (F_2, 20_ = 4.51, *p* < 0.05). A Newmans-Keuls post hoc test showed that the rats that recalled the memory in context B after receiving hippocampal injection of β-ODN had a significantly lower retention (17.5 ± 2.58 s), compared with SC-ODN-injected controls (227.55 ± 55.03 s, *p* < 0.01). In contrast, when the groups of rats were tested in context A, they all showed a strong memory: retention of β-ODN-injected rats was similar to that of both SC-ODN and β-ODN-injected, non-reactivated controls (316 ± 27.10 s, 351 ± 48.09 s, and 339.50 ± 76.81 s, respectively).

Taubenfeld et al. [14] also showed that, like C/EBPβ, protein synthesis in the hippocampus is not required for IA reconsolidation, although it is necessary for its initial consolidation [14,15]. Thus, we determined whether, similarly, memory for context B was dependent upon hippocampal protein synthesis. Rats underwent IA training in context A and reactivation in context B as described above. Immediately after reactivation, they were divided into two subgroups, one of which received bilateral hippocampal injections of the protein synthesis inhibitor anisomycin, while the other received vehicle solution. To exclude any effect on retention of the old memory, additional control groups received anisomycin injections at the same time point after training in the absence of reactivation. All groups of rats were tested 48 h later, first in context B and then in context A. As depicted in Figure 4C, hippocampal injection of anisomycin completely blocked memory for context B, but did not affect memory for context A. A two-way ANOVA between treatment (anisomycin/vehicle/anisomycin-non-reactivated) and context of testing (A/B) revealed a significant effect of context (F_1, 21_ = 141.4, *p* < 0.0001) and a significant treatment × context interaction (F_2, 21_ = 24.33, *p* < 0.0001). A Newman-Keuls post hoc analysis revealed that the new memory for context B, induced by reactivation through the visual CS in this context, was blocked by hippocampal anisomycin injection (Aniso: 33.37 ± 6.29 s; vehicle: 283.87 ± 51.36 s, *p* < 0.01). However, in the same rats, memory for context A remained unaffected (Aniso: 407.50 ± 43.04 s), and was comparable to memory of context A of rats that received either vehicle injection (vehicle: 330.50 ± 29.57 s) or anisomycin injection without reactivation (426.37 ± 38.53 s). As expected, anisomycin-injected rats that did not undergo reactivation in context B showed no memory for this context (31.87 ± 7.76 s).

Anisomycin injections into the amygdala were not used to dissociate between consolidation or reconsolidation requirements because amygdala protein synthesis is required for both consolidation and reconsolidation of IA [20, and Milekic et al., unpublished data].

These results reveal that consolidation, but not reconsolidation, mechanisms contribute to the formation of an association composed of new information and a reactivated memory.

## Discussion

Together, our results indicate that when an established memory is reactivated in the presence of new information, its reconsolidation process is not essential in linking the reactivated memory with the new information. In fact, these two processes are doubly dissociable.

In particular, we provide evidence that, like previously found with other second-order fear-conditioning tasks [18,21–23], the reactivation of IA memory in a new context (context B, S2) results in the formation of an association that links S2 with the recalled fear originally induced by S1-US. Thus, this task is suitable for investigating whether reconsolidation is necessary to form a new association that links S2 with the reactivated fear. Both the new association and the recalled memory are labile after the reactivation by the light and require protein synthesis for their stabilization, as systemic injections of protein synthesis inhibitors cause the loss of both memories. However, although the recalled memory undergoes reconsolidation, this process occurs independently from the formation of the new association S2-fear. The two processes appear to be mechanistically distinct and, in fact, the inhibition of a molecular mechanism required for memory reconsolidation, namely the expression of C/EBPβ in the amygdala, selectively affects the reactivated memory while leaving the new association intact.

Conversely, the formation of the new association S2-fear appears to be mediated by molecular mechanisms similar to those underlying the initial consolidation of a new memory; indeed, hippocampal inhibition of protein synthesis or C/EBPβ expression selectively disrupts the new S2-fear memory, while it leaves the old memory intact. Furthermore, the amnesia of the new S2-fear association produced by hippocampal disruption of protein synthesis or C/EBPβ does not seem to be caused by a defect in the retrieval process because, during the same testing session, the old memory is normally retrieved. These data support the hypothesis that amnesia displayed after the disruption of consolidation is the result of defective stabilization and not of impaired retrieval [24–26].

The amygdala requirement for C/EBPβ during IA reconsolidation, but not consolidation, is intriguing. Antisense-mediated disruption of C/EBPβ in the amygdala 5 h after IA recall impairs retention at later times, and this effect is contingent upon memory reactivation. By contrast, no memory deficit is produced by the same treatment at the same time point during the initial consolidation. Although our results do not exclude the possibility that during consolidation C/EBPβ may play a role in the amygdala at other time points, they reveal that, soon after 5 h post-training or post-recall, this molecule is differentially engaged in consolidation versus reconsolidation. Hence, this requirement at this time point selectively dissociates the contribution of IA reconsolidation. Previous results from Taubenfeld et al. [14] showed that, conversely, C/EBPβ in the hippocampus is required for the consolidation, but not the reconsolidation, of IA. Thus, it appears that both consolidation and reconsolidation are mediated by similar molecular mechanisms, which are critically required in distinct brain regions. This conclusion is not in contrast with the observation by Lee et al. [27] showing that in contextual fear conditioning, distinct molecular mechanisms are engaged in the hippocampus during either consolidation or reconsolidation. In fact, it is possible that memory consolidation and reconsolidation engage both similar and distinct mechanisms, and that C/EBPβ is an example of a molecule that serves both processes [12]. Additionally, our results support the idea that both consolidation and reconsolidation rely on common long-term plasticity mechanisms [13,28].

Our data, together with the results obtained with cued fear conditioning [29–31], contextual fear conditioning [32], conditioned taste aversion [33,34], and appetitive conditioning [35,36], indicate that the amygdala plays a central role in reconsolidation. Thus, interestingly, the amygdala seems to play a general role in the reconsolidation of incentive learning.

Our findings seem to disagree with those indicating that the amygdala, and in particular amygdala N-methyl-D-aspartate receptors, play a critical role in second-order conditioning paradigms. In fact, a second-order appetitive conditioning is blocked by excitotoxic lesions of the amygdala [38]. Moreover, the selective N-methyl-D-aspartate receptor antagonist DL-2-amino-5-phosphonopentanoic acid (APV) prevents the association between S2 and fear elicited by S1 in second-order fear conditioning, while it enhances the expression of the first-order conditioning [23]. These data may appear to contradict our results showing that the amygdala is required for the reconsolidation, but not the consolidation, of the first-order conditioning, and not needed for the formation of the second-order response. A likely explanation for this apparent discrepancy is that we specifically investigated the requirement for C/EBPβ, which does not represent the sum of the mechanisms and pathways activated in the amygdala during either first-order or second-order conditioning. In other words, the amygdala may very well play an essential role in second-order conditioning, but without critically engaging C/EBPβ at the time points tested in our study. In addition, differences may be due to the different tasks employed, and, finally, an important distinction concerning time of treatment and testing needs to be made between these other studies and ours. While we tested the role of molecular mechanisms involved at late time points (C/EBPβ 5 h after training or recall), after training, and tested the effect at 48 h, the other investigations assessed the involvement of other types of molecules (amygdala N-methyl-D-aspartate receptors) during acquisition, and tested memory retention within 1–2 h post-training. As it has been previously reported, regions involved in the acquisition of the task (whether first-order or second-order conditioning) may not participate in the memory stabilization process [38].

Why does the disruption of C/EBPβ in amygdala not affect IA consolidation, but, according to previous evidence by Berman et al. [20], the inhibition of protein synthesis does? In this work, Berman et al. injected protein synthesis inhibitors into the amygdala immediately after training, while we tested the effect of blocking C/EBPβ 5 h after training. Thus, a possible explanation is that blocking protein synthesis in the amygdala at later times, such as at 5 h after training, has no effect on memory consolidation. In addition, it is also possible that the amygdala protein synthesis required for IA consolidation does not involve the C/EBPβ-dependent pathway.

Conversely, the reconsolidation of IA requires protein synthesis, as anisomycin injected into the amygdala immediately after recall results in amnesia (unpublished data). Nevertheless, we chose not to use this amygdala requisite to dissect the role of reconsolidation or consolidation in our second-order IA conditioning because, as indicated by previous literature discussed above [20], amygdala protein synthesis is also necessary for the consolidation of IA. Thus, such a requirement is not useful to dissociate consolidation from reconsolidation.

Hence, taken together, our results indicate that memory reactivation by recall can mediate two independent processes: the formation of a new association composed of new and old and reactivated information and the reconsolidation of the old memory. These processes occur in an independent fashion, as they can be doubly dissociated on the basis of their molecular requirements in distinct brain regions.

Some authors have suggested that each time a memory is reactivated, a new encoding event occurs [39], and it has been proposed that each reactivation may mediate the incorporation of new information [40,41]. Although more information is necessary to understand the role of these new encoding events and whether they represent the formation of an association between new information and an old, reactivated memory, it is reasonable to believe that these or similar processes are engaged in memory updating. In fact, the process of retrieval itself is likely to have an effect on the memory, and, therefore, reactivated memories may not be identical to the original one but rather would be updated at each retrieval. Our data suggest that the process of memory updating, or at least the formation of an association that links new and reactivated information, occurs without destabilizing the original memory trace, independently from the reconsolidation of the retrieved memory.

In conclusion, the evidence provided in this study is in agreement with the working model described in Alberini [12], in which we discussed the hypothesis that one possible function of memory reactivation is to provide an opportunity for integrating new information into old memories. Our present results build on this model by showing that linking new information to a reactivated memory occurs through molecular mechanisms of consolidation and not by engaging mechanisms of reconsolidation induced by the reactivation event. Such a process does not interfere with the stability of the reactivated memory.

According to our working model [12], the process known as reconsolidation represents a phase of the consolidation process that occurs over a relatively long period and is not restricted to the initial protein-synthesis-dependent phase induced by learning, but also includes a number of subsequent reactivation events, which can be either implicit or explicit. These reactivations are processed by circuits that may overlap but are, at least in part, distinct from those underlying the initial, learning-induced phase. The overall process of memory consolidation is therefore a function of time and number of reactivation events and eventually leads to a memory that is increasingly strengthened, as suggested by the findings that recent, but not old, memories are sensitive to protein synthesis inhibitors administered after reactivation [19,28,42]. Our present findings are consistent with this model. They indicate that the protein-synthesis-dependent phase of reconsolidation is not involved in linking new information with reactivated memories. If the opposite would be true, the integration of new information with reactivated memories would be possible only with recently established, but not older, more stable memories. In conclusion, linking old memories with new information can occur without affecting the stability of previously established memories because they are mediated by consolidation types of mechanisms.

## Materials and Methods

### Animals

Long Evans rats (200–250 g) were used in all experiments. Rats were individually housed and maintained on a 12 h on/12 h off light/dark cycle, and experiments were carried out during the light hour cycle. All rats were allowed free access to food and water.

### IA training

The IA chamber consisted of a rectangular-shaped box, divided into a safe compartment and a shock compartment. The safe compartment was white and illuminated; the shock compartment was black and dark. Foot shocks were delivered to the grid floor of the dark chamber via a constant current scrambler circuit. The apparatus was located in a sound-attenuated, non-illuminated room. During training sessions, each rat was placed in the safe compartment with its head facing away from the door. After a period of 10 s, the door separating the compartments was automatically opened, allowing the rat access to the shock chamber. Latency to enter the shock chamber was taken as a measure of acquisition. The door closed 1 s after the rat entered the shock chamber, and a brief foot shock (0.9 mA for 2 s) was administered to the rat. The rat was then removed from the apparatus and returned to its home cage. Retention tests, which also recalled and reactivated the memory, were performed 48 h (Test 1) later by placing the rat back in the safe compartment and measuring the latency to enter the shock chamber. Foot shock was not administered on the retention test, and testing was terminated at 540 s. 48 h after Test 1, animals were retested for retention (Test 2). Latency to re-enter the dark compartment was taken as memory retention. An experimenter blind to the treatment of the groups scored latencies. Statistical analysis of the behavioral data was performed using a one- or two-way ANOVA followed by Student Newman-Keuls post hoc tests. All protocols complied with the National Institutes of Health Guide for the Care and Use of Laboratory Animals and were approved by the Mt. Sinai School of Medicine Animal Care Committees.

### Second-order conditioning IA

A slightly modified protocol of IA and two different shuttle boxes (context A and context B) were used for the behavioral experiments. Each box consisted of two rectangular-shaped Perspex chambers separated by a sliding door. In context A, one chamber was white with grid floor (safe compartment) and the other dark and black (shock compartment) with grid floor. In context B, the safe compartment was very different than that of context A and had a smooth plastic floor and black-and- white striped wallpaper on the walls and roof; the black compartment was the same as in context A. Some perfume was vaporized to make the odor of the context B different. Furthermore, context B was conducted in a different experimental room. During training, rats were placed into context A. A light was switched on 2 s later (visual CS), and 10 s later the door between the two chambers was opened to allow the rat to enter the dark chamber. Here, a foot shock (0.9 mA) was delivered to the grid floor via a constant current scrambler circuit. Rats were then immediately returned to their home cage. 2 d later, rats that received a reactivation trial (context B + light) were placed in context B, and 2 s later the light was switched on. The rats remained in this context for 90 s and were not allowed to enter the dark compartment. Finally, the rats were returned to their home cages. Control groups included rats that were either exposed to context B for 90 s without the light (context B), or rats that did not undergo reactivation and were left in the home cage (non-reactivated). Rats were tested 2 d later, in either context A and/or context B, as detailed in the Results section. No light was switched on during the test session. The latency to enter into the dark (shock) compartment was measured for all groups. Latency to enter the shock chamber before receiving the shock was taken as acquisition. Latency to re-enter the dark compartment was taken as memory retention. An experimenter blind to the treatment of the groups scored latencies.

### Systemic anisomycin administration

Intraperitoneal injections of anisomycin at 210 mg /kg were performed as previously described [14].

### Surgeries

Rats were anesthetized with ketamine (60 mg/kg) and xylazine (7.5 mg/kg), and stainless steel cannulas (22 gauge) were stereotactically implanted bilaterally into either their hippocampi (4 mm posterior to Bregma, 2.6 mm lateral from midline, and 2 mm ventral) or basolateral amygdala nucleus (2.8mm posterior to Bregma, 5.3mm lateral from midline, and 6.25 mm ventral). After surgery, rats were returned to their home cage for a 7-d recovery period.

### Amygdala ODN injections

C/EBPβ antisense (β-ODN; 5′- CCAGCAGGCGGTGCATGAAC-3′) and scrambled oligodeoxynucleotide (SC-ODN; 5′- TCGGAGACTAAGCGCGGCAC-3′) were bilaterally injected in the basolateral amygdala 5 h after training or reactivation (0.5 μl/side; 2 nmol/μl).

### Hippocampal anisomycin and ODN injections

Hippocampal injections of 1 μl of 125 μg/μl of anisomycin (Sigma, St. Louis, Missouri) were performed as previously described. β-ODN or SC-ODN were bilaterally injected in the hippocampi 5 h after reactivation (1μl/side; 2 nmol/μl), as previously described [14].

### Histology

At the end of the experiments, rats were anesthetized, and their brains were removed, frozen, sectioned, and inspected for cannula placement.

## Abbreviations

ANOVA: analysis of variance
CS: conditioned stimulus
IA: inhibitory avoidance
S1: first-order conditioned stimulus
S2: second-order conditioned stimulus
US: unconditioned stimulus
